# Genome-wide analysis and stress-responsive expression of CCCH zinc finger family genes in *Brassica rapa*

**DOI:** 10.1186/s12870-018-1608-7

**Published:** 2018-12-27

**Authors:** Boyi Pi, Xinghui He, Ying Ruan, Jyan-Chyun Jang, Yong Huang

**Affiliations:** 1 College of Bioscience and Biotechnology, Hunan Agricultural University, Changsha, 410128 China; 2 Key Laboratory of Crop Epigenetic Regulation and Development in Hunan Province, Changsha, 410128 China; 3 Key Laboratory of Plant Genetics and Molecular Biology of Education Department in Hunan Province, Changsha, 410128 China; 4 Department of Horticulture and Crop Science, Molecular Genetics, and Center for Applied Plant Sciences, The Ohio State University, Columbus, OH 43210 USA

**Keywords:** CCCH zinc finger family, Tandem CCCH zinc finger, Evolution, Abiotic stress, *Brassica rapa*

## Abstract

**Background:**

Ubiquitous CCCH nucleic acid-binding motif is found in a wide-variety of organisms. CCCH genes are involved in plant developmental processes and biotic and abiotic stress responses. *Brassica rapa* is a vital economic crop and classical model plant of polyploidy evolution, but the functions of CCCH genes in *B. rapa* are unclear.

**Results:**

In this study, 103 CCCH genes in *B. rapa* were identified. A comparative analysis of the chromosomal position, gene structure, domain organization and duplication event between *B. rapa* and *Arabidopsis thaliana* were performed. Results showed that CCCH genes could be divided into 18 subfamilies, and segmental duplication might mainly contribute to this family expansion. C-X_7/8_-C-X_5_-C_3_-H was the most commonly found motif, but some novel CCCH motifs were also found, along with some loses of typical CCCH motifs widespread in other plant species. The multifarious gene structures and domain organizations implicated functional diversity of CCCH genes in *B. rapa*. Evidence also suggested functional redundancy in at least one subfamily due to high conservation between members. Finally, the expression profiles of subfamily-IX genes indicated that they are likely involved in various stress responses.

**Conclusion:**

This study provides the first genome-wide characterization of the CCCH genes in *B. rapa*. The results suggest that *B. rapa* CCCH genes are likely functionally divergent, but mostly involved in plant development and stress response. These results are expected to facilitate future functional characterization of this potential RNA-binding protein family in *Brassica* crops.

**Electronic supplementary material:**

The online version of this article (10.1186/s12870-018-1608-7) contains supplementary material, which is available to authorized users.

## Background

Transcription factor (TF), also known as trans-acting factor, refers to a DNA-binding protein that activates or inhibits transcription of genetic information from DNA to mRNA by interacting with specific *cis-elements* of DNA targets [1, 2]. TFs regulate the development of plant vegetative and reproductive tissues, including roots [3], stem xylary fibers [4], fruits [5], and seeds [6], as well as leaf abscission [7], flowering [8, 9], and plant immunity [10]. TFs play vital roles in response to biotic stresses, such as bacterial, fungal, and viral attacks [11], as well as abiotic stresses, including drought, salt, high osmolarity, heat, or cold [12–15].

Zinc finger motif was first found in the *Xenopus* transcription factor IIIA, and zinc finger protein is now regarded as one of the most abundant protein family in eukaryotic genomes. Zinc finger motif was named based on the zinc-binding amino acids and the requisition of zinc ions to stabilize its structure. Zinc finger motif is a small, functional, independently folded domain [16–18]. Zinc finger proteins play a multi-faceted role in numerous biological processes, including DNA recognition, RNA packaging, transcriptional activation or repression, regulation of apoptosis, protein folding and assembly, and lipid binding [18, 19]. Fourteen zinc finger families have been found in plants, in which DNA or protein binding proteins are over-represented [20].

Cys2His2 (C2H2) zinc finger is the most common DNA-binding motif found in eukaryotic transcription factors [21]. By contrast, the ubiquitous CCCH motif is preferentially function in RNA-binding and processing. In mammals, a prototypical tandem CCCH zinc finger protein tristetraprolin (TTP) binds to the *TNFα* (tumor necrosis factor α) ARE (AU-rich element) in 3’ UTR region to destabilize *TNFα* mRNA [22]. In Arabidopsis and rice, different CCCH motifs are characterized by variable number of amino acid spacers between each cysteine and cysteine-histidine (C-X_4–15_-C-X_4–6_-C-X_3_-H) [23]. The CCCH zinc finger proteins usually contain 1–6 CCCH repeated motifs, and C-X_7/8_-C-X_5_-C-X_3_-H is the most abundant motif in Arabidopsis and rice CCCH proteins. Arabidopsis has 68 CCCH protein genes that are divided into 11 subfamilies [23]. AtC3H14 (At1G66810) and AtC3H15 (At1G68200) containing the C-X_8_-C-X_5_-C-X_3_-H-X_18_-C-X_8_-C-X_5_-C-X_3_-H motif are the only two Arabidopsis tandem CCCH zinc finger (TZF) proteins with a conserved TZF motif identical to animal counterparts [24]. AtC3H48 (AT4G25440), AtC3H59 (AT5G40880), AtC3H62 (AT5G49200), and AtC3H63 (AT5G51980), containing one or two CCCH motif(s) of C-X_7_-C-X_4/5_-C-X_3_-H and additional seven WD40 (WD or beta-transducin repeats) domains, are unique to plants [23, 25, 26]. Rice is another model plant for CCCH zinc finger protein research. Rice CCCH zinc finger proteins contain 67 members in 8 subfamilies. Rice CCCH proteins are mainly involved in abiotic stress response and development [23].

Likewise, Arabidopsis CCCH genes are involved in developmental processes [27–31] and various stress responses [32]. Arabidopsis Arginine-rich motif-tandem CCCH Zinc finger (RR-TZF) proteins, characterized by two identical C-X_7–8_-C-X_5_-C-X_3_-H and C-X_5_-C-X_4_-C-X_3_-H motifs separated by 16–18 amino acids, have been studied quite intensively (reviewed by [33, 34]. *AtTZF* genes are induced by Abscisic acid (ABA), Gibberellin acid (GA), salt, cold, H_2_O_2_, osmotic stress, nutrient deficiency, and mainly involved in growth and stress responses. For example, *AtTZF1* (AtC3H23, At2g25900) is localized in cytoplasmic processing bodies (PBs) and stress granules (SGs) and is positively regulated by ABA, sugar depletion, and salt stress, but negatively regulated by GA. AtTZF1 binds both RNA and DNA in vitro [34–37].

Chinese cabbage (*Brassica rapa* L. ssp*. Pekinensis*) is a subspecies of *B. rapa* (AA, 2n = 20), and one of the most important vegetables in Asia [38]. The genomic organization and function of CCCH genes in *Brassica* species remain uncharacterized. AtC3H36 (AT3G12130) homologous gene of *Brassica napus* CL1Contig3630 is co-expressed in pollen, microspore, ovule and zygotic embryo [39]. A genome-wide transcriptome analysis showed that two CCCH genes participated in dehydration stress response of *B. rapa* [40]. With the advent of genome sequencing technologies, global identification of gene families becomes a reality for numerous plant species. The genomic characterization of CCCH gene families have been carried out in Arabidopsis, rice [23], maize [20], citrus [41], tomato [42], grape [43], poplar [44], alfalfa [45], chickpea [46], *Aegilops tauschii* [47], and switchgrass [48]. Here, we report the genome-wide identification of CCCH genes in *B. rapa* [49] and their expression response to ABA, high temperature, drought, and salt stresses.

## Results

### Identification of CCCH genes in *B. rapa*

**Fig. 1:**
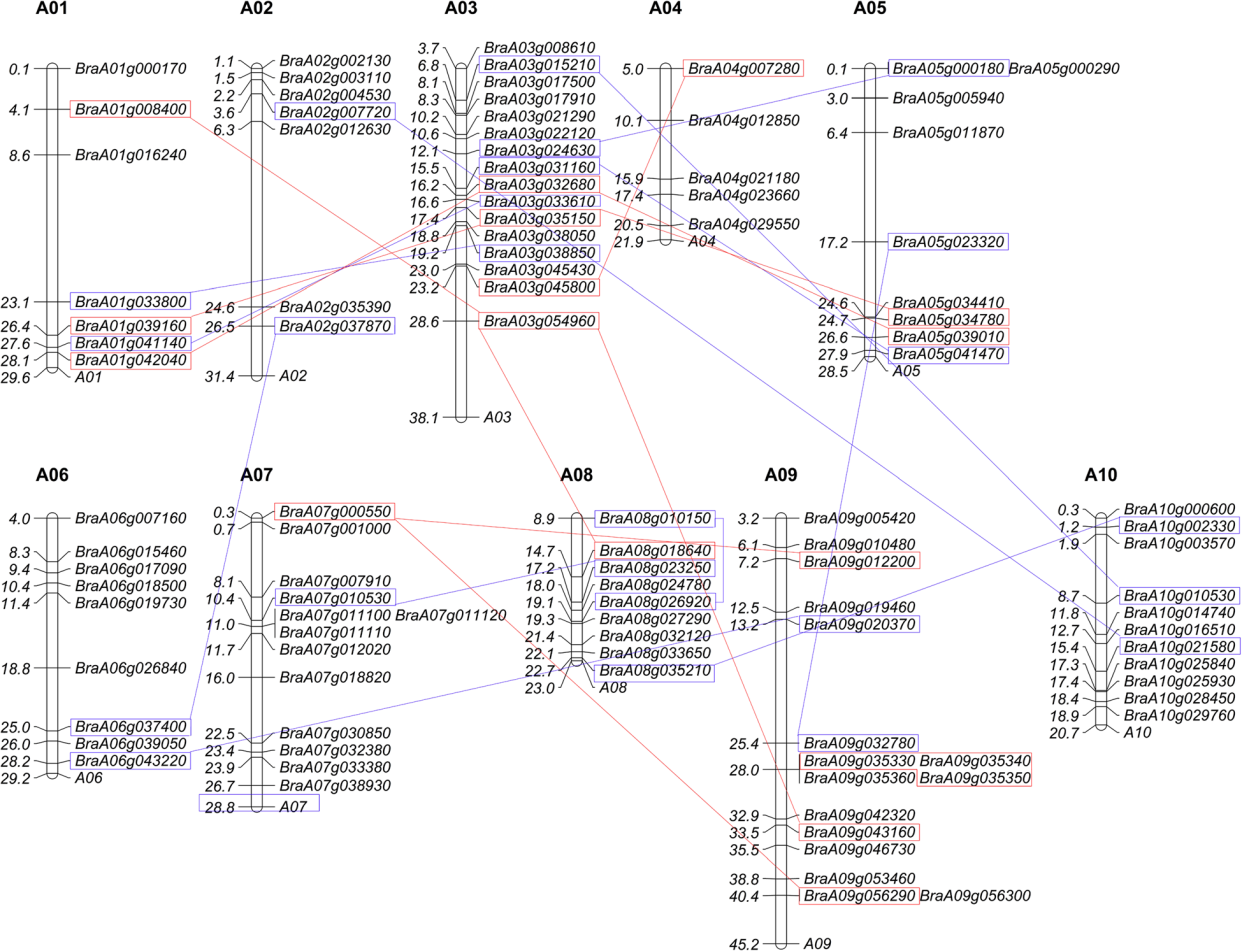
Chromosomal location of *CCCH* genes in *B. rapa.* 102 of the 103 CCCH genes have been mapped on chromosomes A01-A10. The chromosome map was constructed using the Mapchart 2.2 program. The scale on the chromosome represents megabases (Mb) and the chromosome number is indicated at the top of each chromosome. Blue line and rectangle shows diploid gene pairs and red line and rectangle shows triploid gene pairs. All duplicated genes are segmental duplication except *BraA09g035330*/*BraA09g035340*/*BrA09g035350*

**Fig. 2:**
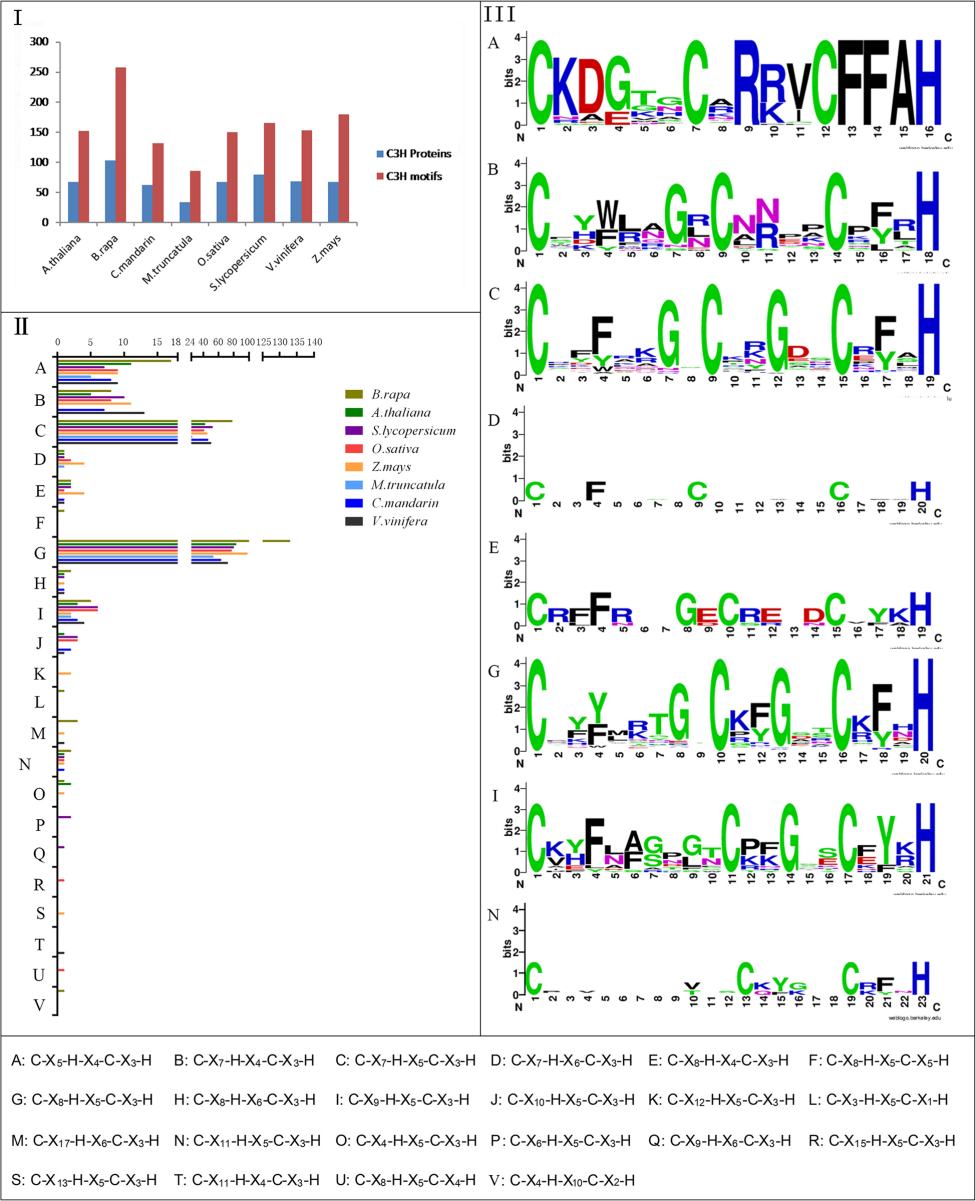
Statistics on the CCCH proteins from 8 plant species. I, Statistics on the amount of CCCH proteins and CCCH motifs from 8 plant species; II, Statistics on CCCH motifs types from 8 plant species; III, Sequence logos for the CCCH motifs in *B. rapa*. Plant species: *Arabidopsis thaliana*, *Brassica rapa*, *Clementine mandarin*, *Medicago truncatula*, *Oryza sativa*, *Solanum lycopersicum*, *Vitis vinifera*, *Zea mays*

Using Arabidopsis CCCH zinc finger proteins as queries, we screened *B. rapa* genome by BLASTp tool (http://brassicadb.org/brad/). We identified and reaffirmed 103 CCCH zinc finger proteins by SMART and NCBI in *B. rapa* (Additional file 1). 102 of the 103 corresponding CCCH genes were mapped to chromosome A01-A10 (Fig. 1). Chromosome A09, the first longest chromosome and Chromosome A03, the second longest chromosome, possesses the largest number (16) of CCCH genes followed by ChrA07 with 13 members. ChrA10 is the shortest chromosome, 20.72 M, and carries the highest density of 11 CCCH genes. ChrA02, the third longest chromosome, carries the lowest density of CCCH genes. No CCCH genes were observed in the middle region (longer than 10 M) of ChrA01 and ChrA02. The average density of CCCH genes on each chromosome is lower than that of Arabidopsis, whereas higher than that of rice and maize (Fig. 2).

### Duplication of CCCH genes in *B. rapa*

Gene duplication plays a vital role in the evolution of new gene functions and is one of the primary drivers of adaptive evolution [50]. According to the criteria established previously, some *B. rapa* CCCH genes have been apparently duplicated [51]. For example, eleven gene pairs are duplicated: *BraA03G024630/BraA05g000180*, *BraA03g031160/BraA05g041470*, *BraA03g015210/BraA10g010530*, *BraA05g023320/BraA09g032780*, *BraA01g033800/BraA03g038850*, *BraA09g020370/BraA06g043220*, *BraA10g002330/BraA08g035210*, *BraA07g010530/BraA08g023250*, *BraA03G033610/BraA01g041140*, *BraA08g026920/BraA08g010150*, *BraA06g037400/BraA02g037870,* whereas six gene groups are shown in triplicate: *BraA03g008610*/*BraA02g007720*/*BraA10G021580*, *BraA03g035150*/*BraA05g034780* /*BraA01g039160*, *BraA03g054960*/*BraA08g018640*/*BraA01g008400*, *BraA03g045800*/*BraA09g043160*/*BraA04g007280*, *BraA09g035330*/*BraA09g035340*/*BraA09g035350*, *BraA09g056290/BraA07g000550*/*BraA09g012200*, and one gene group tetraploid *BraA05g039010/BraAnng001760/BraA03g032680*/*BraA01g042040.* It is surprising that all these genes are segmental duplication except three genes on ChrA09 (Fig. 1; Additional file 2). The highest frequency of CCCH gene segmental duplication events occurred between ChrA01 and ChrA03 which contained five segmental duplication events, followed by four segmental duplication events between ChrA03 and ChrA05. Most of the duplicated gene pairs are linked, suggesting that chromosome or segment duplication might occur among ChrA01, ChrA03 and ChrA05 (Fig. 1). Six additional CCCH gene pairs located on ChrA03 and ChrA05 were not regarded as duplicated genes because CDS coverage or protein identify did not meet the criteria of Yang [51] and Sun [52], even though they are orthologous to the corresponding Arabidopsis genes (Additional file 2). All of the Ka/Ks ratios were significantly < 0.5, suggesting a result of negative selection. The gene duplication date was estimated around 0.29–19.58 MYA (Million Years Ago) (Additional file 2).

### Phylogenetic analysis of CCCH zinc finger proteins in *B. rapa*

**Fig. 3:**
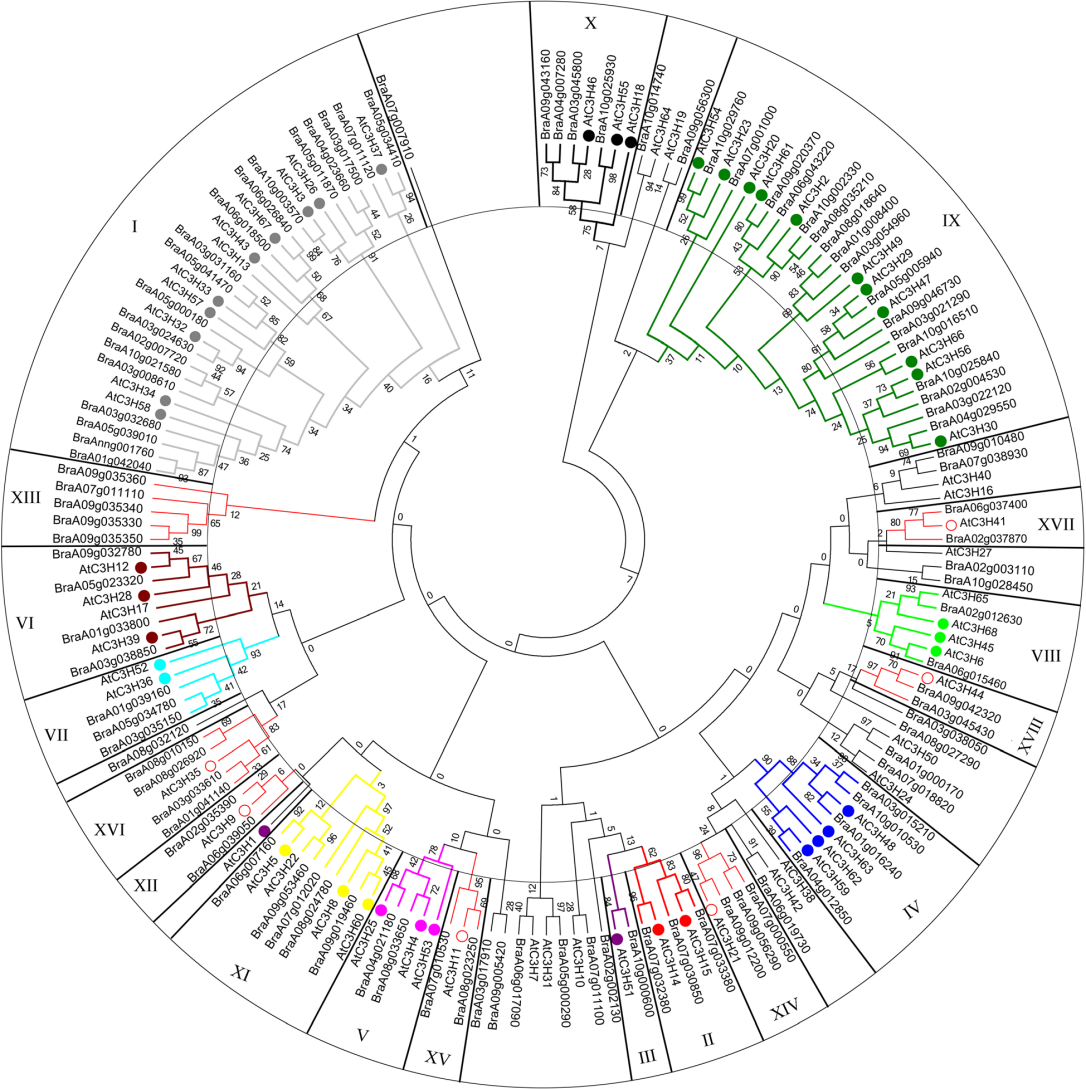
Phylogenetic tree of CCCH proteins in *B. rapa.* This tree includes 103 CCCH proteins from *Brassica rapa and 68 from Arabidopsis thaliana.* 84 of CCCH proteins in *B. rapa* can be grouped into 18 subfamilies and subfamily I-XI are based on reference [23], and subfamily XI-XVIII are new set. Solid circle shows the Arabidopsis CCCH proteins in subfamily I-XI. Protein sequences were aligned using MUSCLE, and the phylogenetic tree analysis was performed using MEGA 7.0. The tree was constructed with the following settings: Statistical Method as Maximum Likelihood; Include Gaps/Missing Data Treatment: Partial deletion; Substitution Model: Poisson model; and Bootstrap test of 1000 replicates for internal branch reliability

**Fig. 4:**
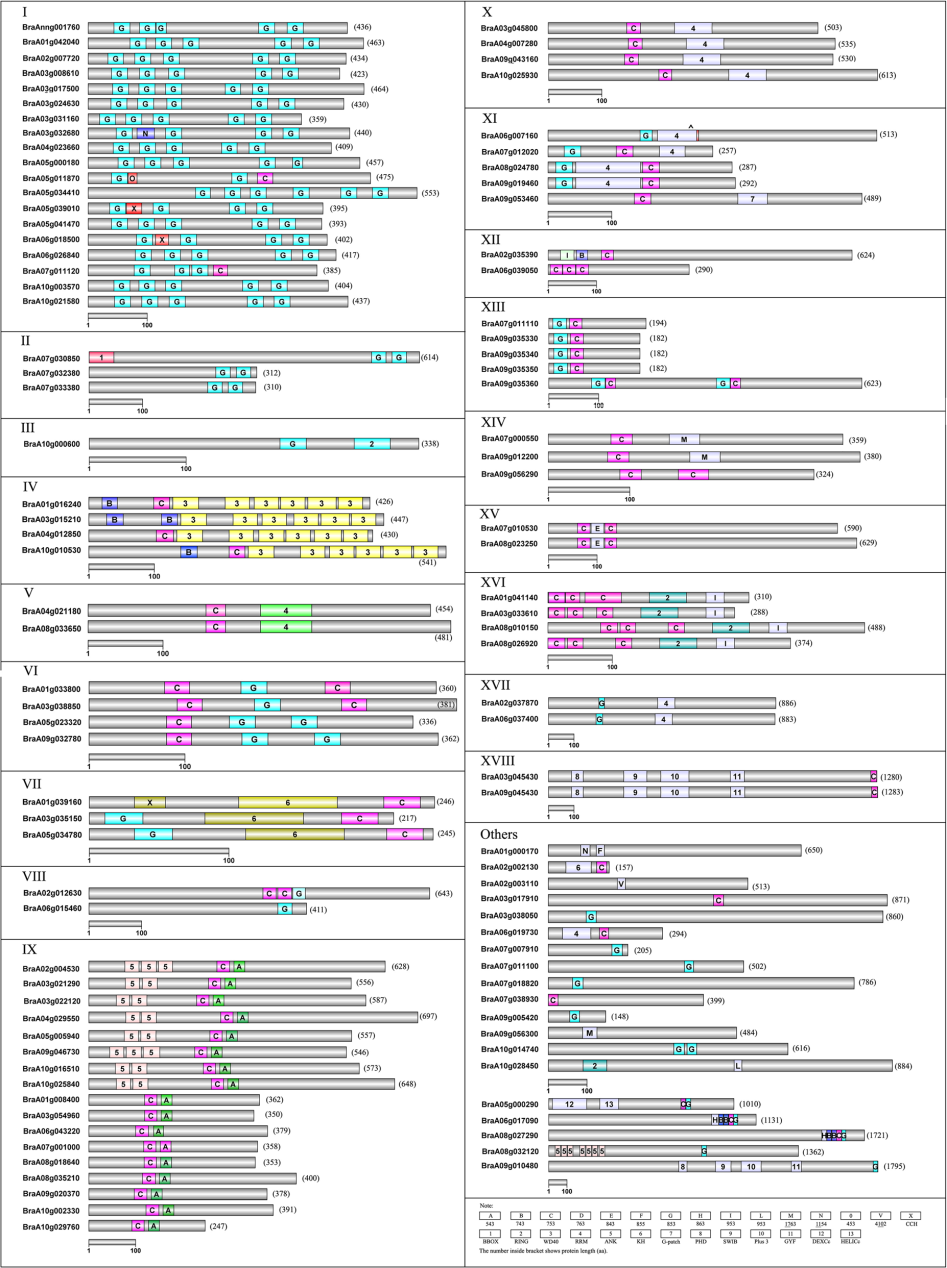
Domain organization of CCCH proteins in *B. rapa.* Domain organization of CCCH proteins were detected by SMART and NCBI (http://www.ncbi.nlm.nih.gov/Structure/cdd/wrpsb.cgi), and the low-complexity filter was turned off, and the Expect Value was set at 10. The site information of domains was subjected to IBS1.0 to construct the proteins organization sketch map

To further characterize the CCCH genes in *B. rapa*, 103 CCCH zinc finger protein sequences were subjected to phylogenetic analysis by the Maximum Likelihood (ML) method (Fig. 3; Additional files 1 and 3; [23]). Based on the Arabidopsis classification system [23], the aforementioned 84 out of 103 CCCH zinc finger proteins could be grouped into 18 distinct subfamilies. Subfamily-I with 19 members is the largest, followed by subfamily IX containing 17, whereas subfamily-III has just one member. Subfamily-XVIII contains the proteins with the longest sequences (Fig. 4). The distribution of CCCH zinc finger proteins in *B. rapa* phylogenetic tree constructed by the Maximum Likelihood method is similar to that made by Neighbor-joining (NJ) phylogenetic trees of Arabidopsis-*B. rapa* (Additional file 4) or ML phylogenetic trees of Arabidopsis-*B. rapa*-rice (Additional file 5).

### Structures of *B. rapa* CCCH genes

**Fig. 5:**
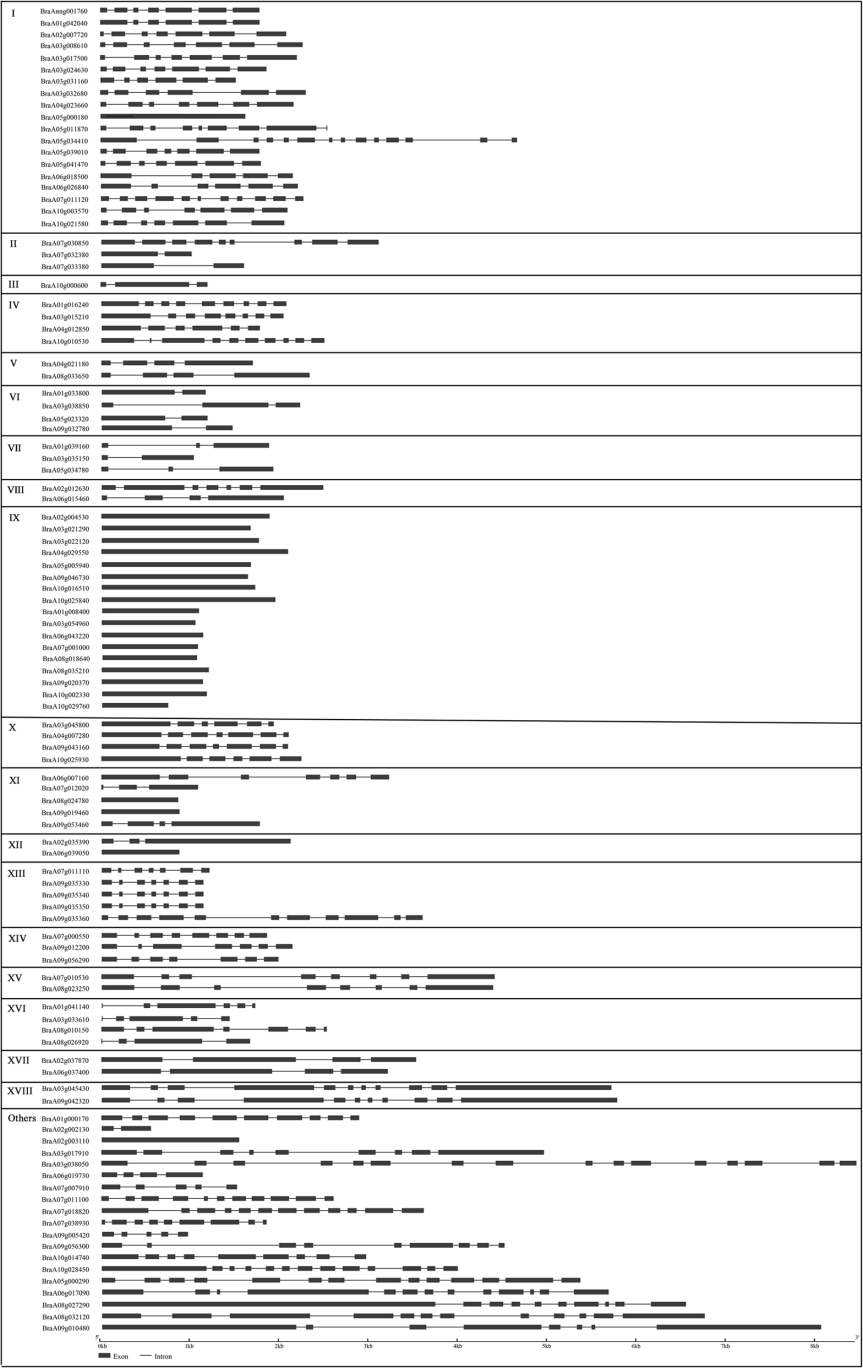
Gene structure of *CCCH* genes in *B. rapa.* The gene structure of 103 CCCH genes was constructed by Gene Structure Display Server (http://gsds.cbi.pku.edu.cn/)

To understand the evolution of gene diversification, the gene structures were analyzed (Fig. 5). Results show that the intron/exon number of CCCH genes changed in a wide range, from1–16, but each subfamily was relatively conserved in gene structure. For example, subfamily-I genes always contain five to seven exons except *BraA05g011870, BraA05g034410 and BraA07g011120*. Subfamily-VI and subfamily-VII genes contain two and three exons, respectively. All gene structures of subfamily-IX have only one exon, not intron. Interestingly, some ungrouped genes had more complex gene structures. Duplicated gene pairs were mainly distributed in subfamily-I and IX, but all of subfamily-VII/XIV members were triplicated genes. Most of the duplicated genes displayed similar gene structure between or among sister genes.

### Domain organization of *B. rapa* CCCH zinc finger proteins

In order to determine the evolution and conservation of CCCH zinc finger proteins, full-length proteins were used to decipher the domain organization. There were significant differences in domain organization among subfamilies, even for the type and number of CCCH motif in *B. rapa*. Altogether, 15 types and 257 CCCH motifs (C-X_3–17_-C-X_4–10_-C-X_1–5_-H) were identified. Among them, C-X_7/8_-C-X_5_-C_3_-H was the most common motif (Figs. 2 and 4). In general, each CCCH zinc finger protein carries 1–6 copies of CCCH motifs (Fig. 4). C-X_7/8_-C-X_5_-C_3_-H motif was mainly found in subfamily-I, VI, VII, VIII, XI and XIII. In contrast to Arabidopsis, C-X_7_-C-X_6_-C-X_3_-H and C-X_10_-C-X_5_-C-X_3_-H motifs were not present in *B. rapa,* whereas novel motif C-X_17_-C-X_6_-C-X_3_-H was found in BraA07g000550 and BraA09g012200 of subfamily-XIV. The C-X_17_-C-X_6_-C_3_-H motif is not present in Arabidopsis, but does exist in maize genome [20]. The novel motifs C-X_3_-C-X_5_-C_1_-H, C-X_17_-C-X_6_-C_3_-H, C-X_8_-C-X_5_-C_5_-H and C-X_4_-C-X_10_-C_2_-H exist in unassorted proteins BraA10g028450, BraA09g056300, BraA01g000170 and BraA02g003110, respectively. Except CCCH motifs, WD40 (WD or beta-transducin repeats), ANK (Ankyrin repeats), RRM (RNA recognition motif) and RING (Really Interesting New Gene) domains exist in subfamily- IV, IX, X/XI, XVI, respectively.

### Conserved stress-responsive subfamily-IX members

**Fig. 6:**
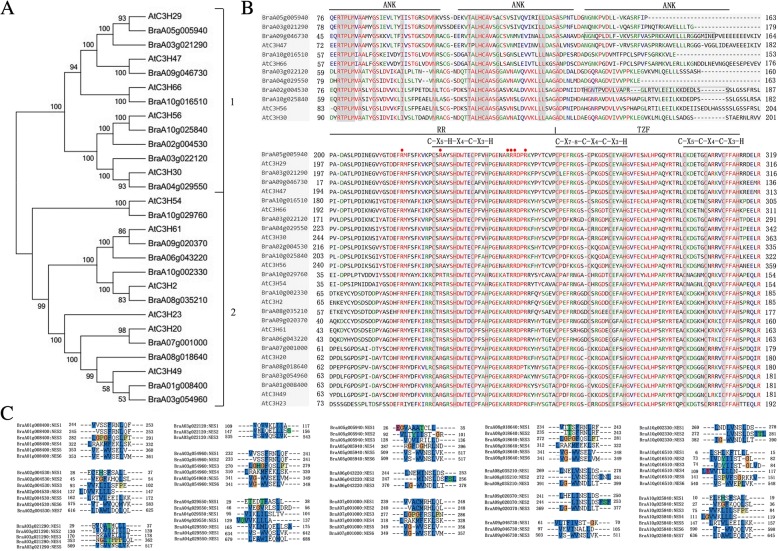
Protein sequence analysis of subfamily-IX in *B. rapa.* **a**, Phylogenetic tree of subfamily-IX proteins in Arabidopsis- *B. rapa* (Statistical Method: Neighbor-joining; Model/Method: No. of differences; Gaps/Missing Data Treatment: Complete deletion; No. of Bootstrap Replications:1000); **b**, Multiple sequence alignment of subfamily-IX proteins in Arabidopsis-*B. rapa*. The domain/motifs are show as on the top; **c**, Amino acid sequence alignment of putative NES sequences of subfamily IX in *B. rapa*

**Fig. 7:**
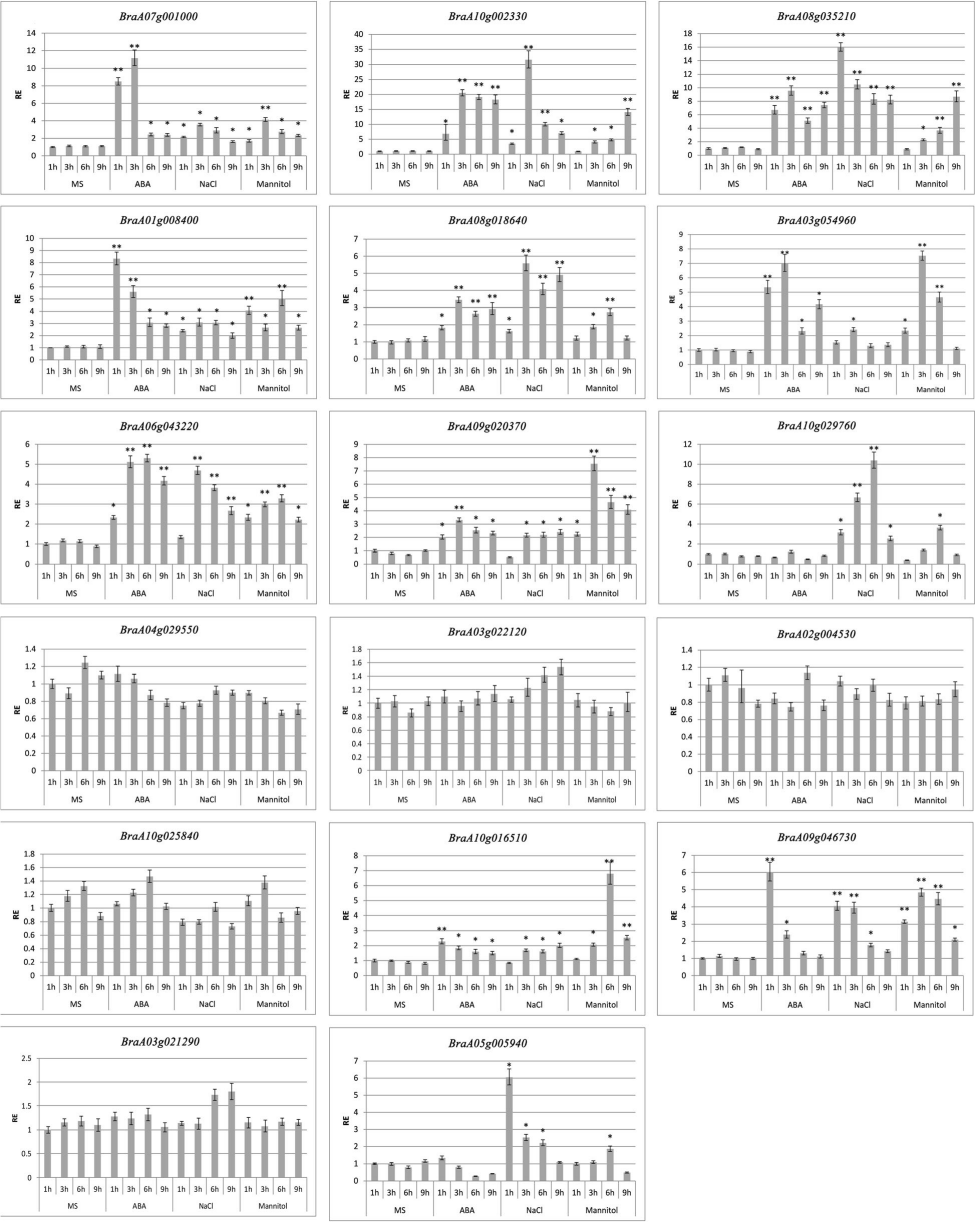
The stress tolerance of subfamily-IX in *B. rapa.* Expression patterns of the subfamily-IX genes under abiotic stress conditions: ABA (100 μM), NaCl (250 mM), drought (300 mM mannitol). The actin gene of *B. rapa* was used as an internal reference. The data are representative of three independent experiments

Arabidopsis RR-TZF genes (Subfamily-IX) play pivotal roles in plant growth, development and stress response likely by targeting AU-rich RNA elements at 3’ UTR and recruiting catabolic machineries to trigger mRNA degradation [34]. To characterize the corresponding subfamily-IX homologs in *B. rapa*, the proteins sequences were used to constructing the phylogenetic tree and deciphering the domain organization, and their transcriptional responses to ABA, drought, and salt stresses were determined (Figs. 6 and 7).

Arabidopsis and *B. rapa* RR-TZF proteins can be divided into two groups (Fig. 6a), due to highly conserved Ankyrin repeats (ANK domain) which is essential for all known Notch signaling pathway by mediating protein-protein interactions [53]. Group-2 members contain both arginine-rich motif (RR) and TZF domains, whereas group-1 members contain an additional two or three ANK repeats. *B. rapa* RR*-*TZF proteins contain conserved C-X_7–8_-C-X_5_-C-X_3_-H and C-X_5_-C-X_4_-C-X_3_-H motifs spaced by 16 amino acids and an RR motif which contains a conserved C-X_5_-H-X_4_-C-X_3_-H motif (Fig. 6b). We used a program from Wang et al. [23] to detect Nuclear Export Signal (NES) in *B. rapa* RR-TZF proteins, and found all members contained a putative NES sequence, indicating that they may be nucleocytoplasmic shuttling proteins involved in signal transduction (Fig. 6c).

The expression profiles of *RR-TZF* genes in *B. rapa* leaves at 4 different developmental stages are detected by real-time PCR (Fig. 7). Twelve of the 17 RR-TZF genes showed remarkably elevated expression under NaCl, ABA or mannitol treatment. However, *BraA02g004530, BraA03g021290, BraA03g022120, BraA04g029550, BraA10g025840* showed no significant expression changes, so they might not be involved in responses to these stresses. Most of the RR-TZF genes had much higher expression under ABA than under control (MS) conditions, except for the five aforementioned genes and *BraA10g029760* and *BraA05g005940.* Among the ABA-responsive genes, *BraA07g001000*, *BraA01g008400*, *BraA03g054960* and *BraA09g046730* showed remarkable induction, with higher expression levels than under NaCl or mannitol stress stimulus. *BraA10g029760* and *BraA05g005940* were easily induced by NaCl, while *BraA09g020370* was strongly induced by mannitol stress. Most *B. rapa* RR-TZF genes showed increasing expression within 3–6 h, after which their expression declined, except for *BraA01g008400* and *BraA09g046730* under ABA stress, and *BraA08g035210* and *BraA05g005940* under NaCl stress, which showed expression peaks after only 1 h.

## Discussion

### Identification and classification of *B. rapa* CCCH zinc finger proteins

Brassica crops are not only vital economic crops, but also classical model plants of polyploidy evolution. *B. rapa*, one of the most important vegetable crops and genomic model organisms, may be the putative contributor of the A-subgenome (*B. oleracea* provides C-subgenome, and *B. napus*, the hybrid offspring *B. rapa* and *B. oleracea*, contains A- and C-subgenome) [49]. Previous studies showed that transcription factors of *B. rapa* were significantly over-retained [49]. CCCH genes, as ubiquitous regulators in a variety of organisms, function in plant development and stress response by interacting with DNA, RNA or proteins [22, 23]. Rameneni et al. has been just identified 63 CCCH genes in *B. rapa* [54], and it was much less than its expected quantity [49]. Here, we identified and confirmed 103 CCCH genes in *B. rapa* (Fig. 1, Additional file 1), which is much more abundant than 68 in Arabidopsis (*n* = 5, [23]), 67 in rice (*n* = 12, [23], 68 in maize (*n* = 10, [20] and even that of plant species with nearly twice chromosome number, such as 91 in poplar (*n* = 19, [44]) and 69 in grape (n = 19, [43]), and a similar number to tetraploid switchgrass [48].

Plant CCCH zinc finger motifs display a wide spacing pattern as C-X_4–15_-C-X_4–6_-C-X_3_-H [23]. As shown in this study, 15 types of CCCH motif are found in *B. rapa* (Figs. 2 and 4). Similar to other plant species, the C-X_7/8_-C-X_5_-C-X_3_-H motif is the most abundant pattern (82.10%), which is comparable to Arabidopsis (82.24%), maize (79.44%), and rice (78.67%) (Additional file 6). Compared to other plant species, some CCCH motifs are missing while other novel CCCH motifs are evolved in *B. rapa*. For example, compared with Arabidopsis, C-X_10_-C-X_5_-C-X_3_-H and C-X_7_-C-X_6_-C-X_3_-H motif is not detected in *B. rapa*, whereas novel C-X_17_-C-X_6_-C-X_3_-H, C-X_8_-C-X_5_-C-X_5_-H, C-X_4_-C-X_10_-C-X_2_-H and C-X_3_-C-X_5_-C-_1_-H motifs are found; compared with rice, *B. rapa* has additional C-X_/8/17_-C-X_6_-C-X_3_-H, C-_4_-C-X_5_-C-X_3_-H C-X_8_-C-X_5_-C-X_5_-H, C-X_4_-C-X_10_-C-X_2_-H and C-X_3_-C-X_5_-C_1_-H motifs, but lacks the C-X_10/15_-C-X_5_-C-X_3_-H C-X_8_-C-X_5_-C-X_4_-H and C-X_7_-C-X_6_-C-X_3_-H motif [23]; compared with maize, the C-X_8_-C-X_5_-C-X_5_-H, C-X_4_-C-X_10_-C-X_2_-H and C-X_3_-C-X_5_-C_1_-H motif are novel in *B. rapa*, but C-X_12/13_-C-X_5_-C-X_3_-H and C-X_7_-C-X_6_-C-X_3_-H motif are not found [20]. These results suggest that the conserved C-X_7/8_-C-X_5_-C-X_3_-H motif plays a crucial functional role in the CCCH protein family. Except for the CCCH motifs, a plethora of other regulatory domains such as RING, WD40, RRM, and KH (K homology motif), are also found in *B. rapa* CCCH proteins (Fig. 4). It is interesting that most of these additional domains are involved in the interaction with RNA, DNA or proteins [28, 55–60].

*B. rapa* genes have an average transcript length of 2015 bp, a coding sequence length of 1172 bp and a mean of 5.03 exons per gene [49]. *B. rapa* CCCH genes intron/exon numbers varies in a wide range, from1–16. Most of the sequences have less than 10 exons, and the average is 5.72 exons per gene (Fig. 5). Moreover, similar to maize, the range of exon length of *B. rapa* CCCH genes is very wide. Together these results indicate that the gene structure and function are highly diverse among CCCH genes [43]. However, the gene structure and domain organization are relatively conserved in each subfamily, suggesting functional redundancy of the subfamily members.

### Duplication and evolution of *B. rapa* CCCH genes

The CCCH family appears to undergo complicated evolution processes and become one of the largest gene families in plants [23, 44]. *Brassica* genome has undergone an additional whole genome triplication (WGT) since its divergence from the Arabidopsis lineage at least 13–17 MYA. More than 90% of the *B. rapa* genome is syntenic with that of the *Arabidopsis* genome [49]. Previous study also showed that the triplicated *Brassica* genome segments diverged from a common ancestor soon after Arabidopsis and *Brassica* lineages divergence, and about 35% of these genes have been lost, most likely via a deletion mechanism in an interspersed pattern [61]. However, genes encoding proteins involved in signal transduction or transcriptional regulation are largely well retained [49, 61]. Environmental factors may play crucial roles in transcription factors reservation [49]. Similarly, CCCH gene family of *B. rapa* may have expanded because of genome triplication. After that the gene family might still retain most key members because of their comprehensive and vital functions in response to abiotic or biotic stresses, although there is an overall shrinkage of some members since the total number is just ~ 1.5 fold to that of Arabidopsis ([49], Fig. 2). *Brassica* species *B. oleracea* (2n = 20) and *B. nigra* (2n = 16) CCCH families also expanded, possessing 75, 92 sequences respectively (Additional files 3, 7 and 8).

It is surprising that all 17 duplicated CCCH gene pairs are segmental duplication and except one group on ChrA 09, and all sister genes are orthologous of Arabidopsis counterparts except for one pair (Fig. 1; Additional file 2). Ka, Ks, and Ka/Ks ratios of duplicated genes were calculated to explore the mode of selection [20, 62]. Generally, a ratio < 1 means negative selection, a ratio = 1 means neutral selection, while a Ka/Ks ratio > 1 means positive selection [20]. Most of the resultant Ka/Ks ratios were significantly < 0.5 (Additional file 2). These results are highly consistent with previous reports, strongly suggesting that most of duplicated CCCH genes undergo purifying selection and the functions of the duplicated genes don’t diverge much after the duplication events [20, 44]. The relative conserved gene structures and absolute conserved domain organizations in/among sister genes of duplicated gene pairs further demonstrate the retentive function (Figs. 4 and 5).

The estimated dates of duplication indicated that 9 gene-pairs duplication events might occur before the additional whole genome triplication (WGT), and the others might occur after that (Additional file 2; [49]).

### Domain organization diversity and putative function analysis

Domain organization directly related to the protein functions [63]. Domain organization is conserved between Arabidopsis and *B. rapa.* Most of subfamily-I proteins are highly conserved with five C-X_8_-C-X_5_-C-X_3_-H motifs, such as Arabidopsis AtC3H37 (HUA1, AT3G12680) that regulates stamen, carpel, and floral development by preferentially binding to poly rU and poly rG [27]. Subfamily-II is conserved between plant and animal, such as Arabidopsis AtC3H14 (At1G66810) and AtC3H15 (At1G68200) containing the typical C-X_8_-C-X_5_-C-X_3_-H-X_18_-C-X_8_-C-X_5_-C-X_3_-H motif found in animal TZFs. AtC3H14 is involved in secondary wall biosynthesis [24]. Subfamily-III such as Arabidopsis AtC3H1 (AT1G01350) with C-X_8_-C-X_5_-C-X_3_-H motif also belongs to RING-finger family, and is involved in lignin biosynthesis [28, 29]. The *B. rapa* subfamily-III homolog BraA10g000600 has conserved CCCH and RING domain. All subfamily-IV proteins, including Arabidopsis and *B. rapa* homologs, contain a C-X_7_-C-X_4_-C-X_3_-H or C-X_7_-C-X_5_-C-X_3_-H motif, plus additional six WD40 repeats, which is a protein-protein or protein-DNA interaction domain [56]. The subfamily-IV proteins are plant-specific and may be involved in chlorophyll biosynthesis and light response [23, 25]. subfamily-V, X and XI proteins in Arabidopsis contain a C-X_7_-C-X_5_-C-X_3_-H motif and an RNA recognition motif (RRM). RRM is the most common RNA-binding domain in eukaryotes. Plant RRM-containing proteins are involved in the regulation of flowering and adaptation to heat stress [57, 58, 64]. Subfamily-VI, BraA05g023320 and BraA09g032780 have one C-X_7_-C-X_5_-C-X_3_-H and two C-X_8_-C-X_5_-C-X_3_-H motifs, whereas the BraA01g033820 and BraA03g038850 have two C-X_7_-C-X_5_-C-X_3_-H and one C-X_8_-C-X_5_-C-X_3_-H motif. Subfamily-VII proteins, containing a C-X_7_-C-X_5_-C-X_3_-H, a C-X_8_-C-X_5_-C-X_3_-H and a conserved RNA-binding K homology motif (KH), are conserved in domain organization and may possess transactivation and RNA-binding activities, that are also known to have redundant roles in the regulation of flowering and senescence in Arabidopsis [31, 59]. Subfamily-IX members are characterized by two identical C-X_7–8_-C-X_5_-C-X_3_-H and C-X_5_-C-X_4_-C-X_3_-H motifs separated by 16–18 amino acids. The subfamily-IX proteins are involved in plant growth, development, and stress response [33, 34]. Subfamily-X and subfamily-XI have similar domain organization. Subfamily-XV proteins have two C-X_7_-C-X_5_-C-X_3_-H and one C-X_8_-C-X_4_-C-X_3_-H. Subfamily-XVI proteins have three C-X_7_-C-X_5_-C-X_3_-H, one C-X_9_-C-X_5_-C-X_3_-H and a protein-binding RING domain. Subfamily-XVII proteins have a C-X_7_-C-X_5_-C-X_3_-H and an auxin-repressed motif. Two subfamily-XVIII proteins carry PHD (Plant Homeo Domain), SWIB (SWI/SNF complex B) and GYF (glycine-tyrosine-phenylalanine) motifs except C-X_7_-C-X_5_-C-X_3_-H. Except CCCH, RING, PHD, RRM, ANK motifs are also responsible for protein-protein or protein-DNA or RNA binding, and it suggests that CCCH protein might involved in multifunction [53, 57, 58, 64, 65].

### The response of *B. rapa* RR-TZF genes to stresses

Arabidopsis and *B. rapa* RR-TZF subfamily proteins might be nucleocytoplasmic shuttling proteins involved in signal transduction (Fig. 6c; [23]). Arabidopsis RR-TZF proteins are involved in growth and stress responses by functioning in signal transduction of ABA, salt, cold, H_2_O_2_, osmotic, and sugar depletion stresses [34, 66]. AtTZF1, AtTZF2 (AtC3H20/AtOZF1, AT2G19810), and AtTZF3 (AtC3H49/AtOZF2, AT4G29190) are highly conserved in various plant species, and regulate seed germination and responses to ABA, Methyl jasmonate (MeJA), GA, oxidative, and salt stresses [34–37]. AtTZF4 (AtC3H2/SOM, At1g03790), AtTZF5 (AtC3H61, At5g44260) and AtTZF6 (AtC3H54/PEI1, AT5g07500) are involved in light, ABA, GA response and modulate seed germination [66–68]. AtTZF7 (AtC3H30, AT2G41900), AtTZF8 (AtC3H56, AT5G12850), AtTZF9 (AtC3H66, AT5G58620), AtTZF10 (AtC3H29/AtSZF2, AT2G40140), and AtTZF11 (AtC3H47/AtSZF1, AT3G55980) are involved in abiotic or biotic stress tolerance responses and stress-induced transition to flowering [34, 69]. There are two copies of AtTZF4 and AtTZF5, and three copies of AtTZF3, but no AtTZF1 orthology found in *B. rapa* (Fig. 6a, Additional file 2). The much similar gene structures and domain organizations of TZF genes between Arabidopsis and *B. rapa* suggest their conserved biological functions (Figs. 4 and 5). The expression patterns of 12 RR-TZF genes of *B. rapa* implicate that they are likely involved in ABA, drought or NaCl stress response (Fig. 7). However, their transcriptional responses to these stress stimuli are different even among the homologous genes. For example, the response of *BraA10g002330* and *BraA08g035210* is similar to the homologous gene *AtTZF4,* but *BraA08g018640* expresses much more than the homologous gene *AtTZF3* under NaCl stress conditions. These results indicate that some of the homologous gene functions might have diverged in *B. rapa*. ABA, a key messenger in plants’ responses to abiotic stresses, is involved in various signaling processes inducing JA-dependent defense response and plant immune response [70]. The expression profiling results show that RR-TZF genes respond to ABA faster than to NaCl and mannitol stress, with the exception of *BraA08g035210*, *BraA08g018640*, *BraA10g029760* and *BraA05g005940* to NaCl stress, and *BraA10g01510* to mannitol stress.

## Conclusion

In this study, we identified 103 CCCH genes in *B. rapa*. Eighty-eight of these genes are categorized into 18 subfamilies based on the results of phylogenetic, gene structure and domain organization analysis. Gene structure and domain organization results reveal that CCCH genes are functional diverged, but highly conserved among members within subfamily. There are nine diploid gene pairs and seven triploid gene pairs, and all duplicated genes are due to segmental duplication. Furthermore, the results of expression profiling suggest that members of subfamily-IX might be involved in ABA, drought, and salt stress response.

## Methods

### Identification of CCCH zinc finger genes and chromosomal map construction

Sequences of Arabidopsis CCCH zinc finger proteins from [23] were used as queries to search the *Brassica rapa* genome (http://brassicadb.org/brad/) with BLASTp tool. All putative CCCH zinc finger proteins were re-confirmed in SMART (http://smart.embl-heidelberg.de/) and NCBI (https://www.ncbi.nlm.nih.gov/Structure/cdd/wrpsb.cgi) in SMART database (version 6.0). The low-complexity filter was turned off, and the Expected Value was set at 10 [71]. The CCCH gene loci information of *B. rapa* (Chromosome version 3.0, 2018, http://brassicadb.org/brad/datasets/pub/Genomes/Brassica_rapa/V3.0/) was used to generate chromosome maps with the Mapchart 2.2 program [72].

### Analysis of gene structure, domain organization, and phylogenetic relationship

The gene structures were visualized using the Gene Structure Display Server (http://gsds.cbi.pku.edu.cn/). The site information of the domain organization was used to construct a protein organization sketch map using IBS1.0 [73].

CCCH motif sequences were extracted from putative CCCH zinc finger proteins, and aligned with ClustalW (http://www.genome.jp/tools/clustalw/), and the resulting files were used to create Logo maps (http://weblogo.berkeley.edu/logo.cgi).

Multiple sequence alignment of CCCH zinc finger proteins was carried out using the MUSCLE (MUltiple Sequence Comparison by Log- Expectation) program [74] and the resulting file was subjected to phylogenic analysis using the MEGA 7.0 program [75]. A tree was constructed based on the full-length protein sequences using the Maximum Likelihood (ML) method with Partial deletion and Poisson model, and a Bootstrap test of 1000 replicates for internal branch reliability.

### Duplicated genes encoding CCCH zinc finger proteins

Duplicated genes were defined according to Yang et al. [51]: gene pairs in which both the coverage of the shorter full-length-CDS sequence covering and the identifies of their encoding amino acid > 70% were regarded as duplicated genes. Tandem duplicates were defined following Sun et al. [52]: duplicated genes located within 100 kb that were separated by ten or fewer non-homologues were defined as tandemly duplicated genes. The full-length CDS sequence coverage and amino acid identities were determined using Blastn/Blastp at the NCBI website [71].

The number of nonsynonymous mutations (Ka) and the number of synonymous substitutions (Ks) of duplicated genes were calculated by DnaSP 6.0 [62]. The Ka/Ks ratios between duplicated genes were analyzed to determine the mode of selection. The duplication time (T, million years ago, MYA) was calculated as T = Ks/2λ × 10^− 6^ (λ = 1.5 × 10^− 8^ for *B. rapa* [76].

### Plant material and stress treatment

*B. rapa* seedlings were grown on 1/2 MS plates at 25 °C under a 16 h light/8 h dark photoperiod. Three-week-old seedlings with 2–3 true leaves were placed in a growth chamber for 3 days to acclimatize before treatment with ABA, NaCl and mannitol. The stress treatments were conducted in accordance with Lee et al. [77]. The whole seedlings were harvested and put into 1/2 liquid MS medium with 250 mM NaCl, 100 μM ABA or 300 mM mannitol. The seedlings were sampled to detect gene expression response to stress at 1, 3, 6, and 9 h and untreated seedlings were used as control at the same time points. Triplicate seedling samples were collected. The materials were quickly frozen in liquid nitrogen and stored at − 80 °C for further analysis.

### RNA extraction and real-time quantitative RT-PCR

RNA extraction and Real-time quantitative RT-PCR were conducted as described previously [71]. Total RNA was extracted from the samples using a TRIzol reagent kit (Invitrogen, Carlsbad, CA, US) according to the manufacturer’s specifications. The RNA integrity was evaluated using agarose gel electrophoresis and ethidium bromide staining. The RNA preparation was then treated with Dnase I and first strand synthesis of cDNA was performed by using oligo (dT) primer and RT Enzyme (Thermo Fisher, USA).

The quantitative real-time PCR was carried out with SYBR-green fluorescence using a CF × 96 Real Time System (BIORAD) with a 20 μl PCR reaction mixture that included 8.8 μl of diluted cDNA, 10 μl of 2 × FastStart Universal SYBR Green Master (ROX) (Roche, Switzerland), and 0.6 μl of forward and reverse primer (Additional file 9). The *BraA02g003190* gene was used as a reference gene. Each sample was run in triplicate for analysis. At the end of the PCR cycles, melting curve analysis was performed to validate the specific generation of the expected PCR product. The expression levels of RR-TZF genes were calculated with the 2 − ^ΔΔCT^ method [78].

## Additional files

Additional file 1: Detailed information of CCCH gene family in *B.rapa*. Sequences and information of CCCH genes and proteins came from http://brassicadb.org/brad/ (XLSX 51 kb)

Additional file 2: CCCH duplicated genes of *B. rapa*. Syntenic analysis between Arabidopsis and *B. rapa* was detected at http://brassicadb.org/brad/searchSyntenytPCK.php; Ka and Ks were calculated by DnaSP 6.0; Cover of CDS and Identify of protein was checked by NCBI. (XLSX 15 kb)

Additional file 3: Detailed information of CCCH gene family in Arabidopsis, rice, *B. oleracea* and *B. nigra.* Information came from Wang et al. [23]. (XLS 238 kb)

Additional file 4: NJ phylogenetic tree of *Arabidopsis*- *B. rapa*. Protein sequences were aligned using ClustalX (1.83) and the phylogenetic tree analysis was performed using MEGA 7.0. The tree was constructed with the following settings: Statistical Method as Neighbor-joining; Include Sites as Partial deletion option for total sequence analyses; Substitution Model: p-distance; and Bootstrap test of 1000 replicates for internal branch reliability. (JPG 1481 kb)

Additional file 5: ML phylogenetic tree of *Arabidopsis-B. rapa*-Rice. 238 CCCH proteins sequences (*Arabidopsis* 68, *B. rapa* 103*,* Rice*,* 67) were aligned using MUSCLE and the phylogenetic tree analysis was performed using MEGA 7.0. The tree was constructed with the following settings: Statistical Method as Maximum Likelihood; Include Sites as Partial deletion option for total sequence analyses; Substitution Model: Poisson model; and Bootstrap test of 500 replicates for internal branch reliability. (JPG 7157 kb)

Additional file 6: The number of different CCCH types in Plants. (XLSX 10 kb)

Additional file 7: NJ phylogenetic tree of *Arabidopsis- B. oleracea.* 143 proteins sequences (*Arabidopsis* 68, *B. oleracea* 75) were aligned using MUSCLE and the phylogenetic tree analysis was performed using MEGA 7.0. The tree was constructed with the following settings: Statistical Method as Neighbor-joining; Include Sites as Partial deletion option for total sequence analyses; Substitution Model: p-distance; and Bootstrap test of 1000 replicates for internal branch reliability. (JPG 623 kb)

Additional file 8: NJ phylogenetic tree of *Arabidopsis- B. nigra.* 160 proteins sequences (*Arabidopsis* 68, *B. nigra 92*) were aligned using MUSCLE and the parameter of phylogenetic tree analysis was same to additional file 7. (JPG 831 kb)

Additional file 9: Primers list of subfamily- IX in *B. rapa.* (XLSX 12 kb)

## Abbreviations

ABA: Abscisic acid
ANK: Ankyrin repeats
ARE: AU-rich element
*B. nigra*: *Brassica nigra*
*B. oleracea*: *Brassica oleracea*
*B. rapa*: *Brassica rapa*
GA: Gibberellin acid
GYF: Glycine-tyrosine-phenylalanine
KH: K homology motif
MeJA: Methyl jasmonate
ML: Maximum Likelihood
MYA: Million Years Ago
NES: Nuclear Export Signal
NJ: Neighbor-joining
PBs: Processing Bodies
PHD: Plant Homeo Domain
RING: Really Interesting New Gene
RR: Arginine-rich
RRM: RNA recognition motif
RR-TZF: Arginine-rich motif-tandem CCCH Zinc Finger
SGs: Stress Granules
SWIB: SWI/SNF complex B
TF: Transcription factor
TNFα: Tumor Necrosis Factor α
TTP: Tristetraprolin
TZF: Tandem CCCH Zinc Finger
WD40: WD or beta-transducin repeats
WGT: Whole Genome Triplication
