# Supplementary figures and images for: Genome-wide analysis and stress-responsive expression of CCCH zinc finger family genes in Brassica rapa
Source: BMC Plant Biol. 2018 Dec 27;18:373. doi: 10.1186/s12870-018-1608-7 (PMC6307296; doi:10.1186/s12870-018-1608-7)

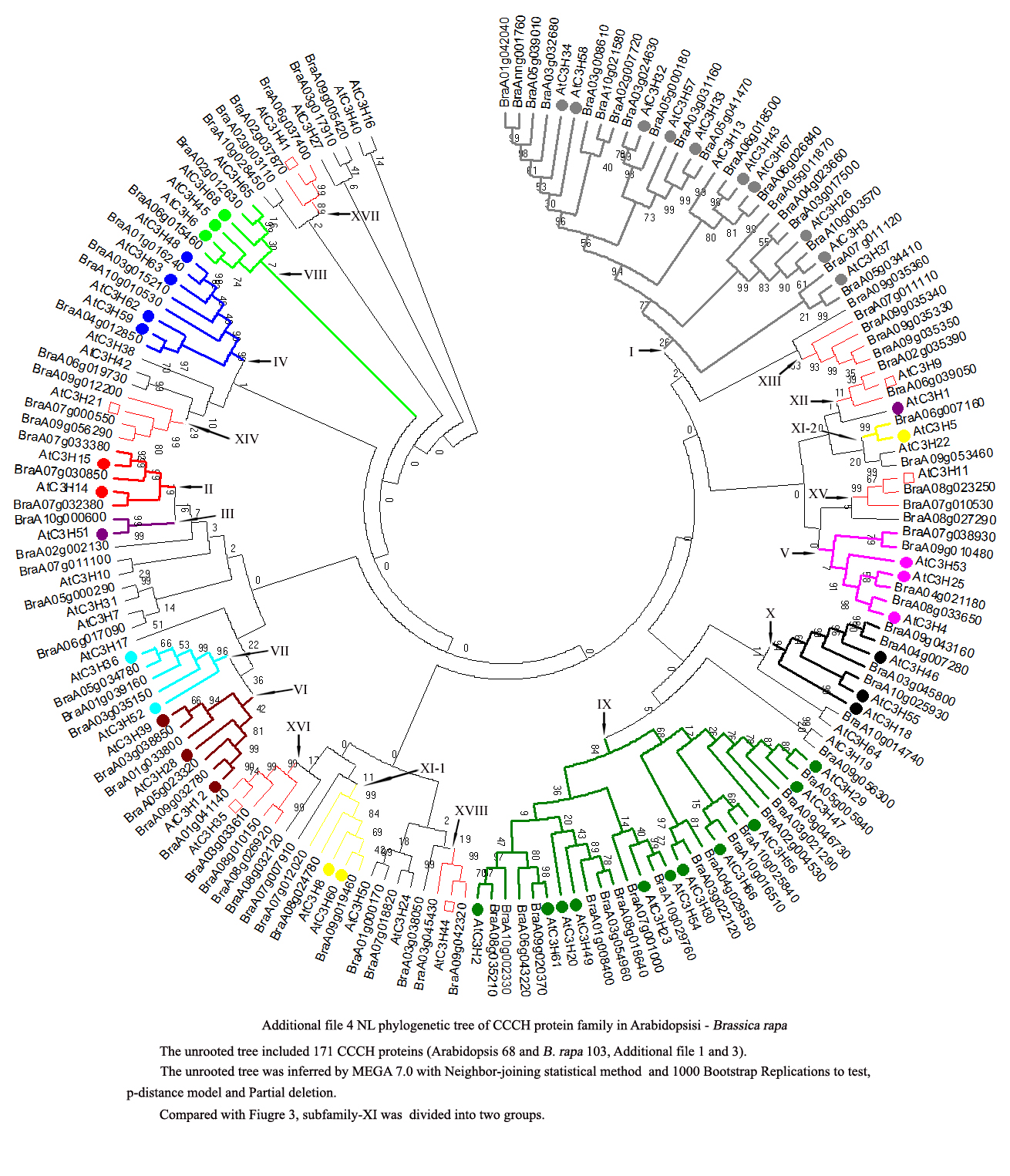

Supplement: Supplementary file 4 — NJ phylogenetic tree of Arabidopsis- B. rapa. Protein sequences were aligned using ClustalX (1.83) and the phylogenetic tree analysis was performed using MEGA 7.0. The tree was constructed with the following settings: Statistical Method as Neighbor-joining; Include Sites as Partial deletion option for total sequence analyses; Substitution Model: p-distance; and Bootstrap test of 1000 replicates for internal branch reliability. (JPG 1481 kb) [file 12870_2018_1608_MOESM4_ESM.jpg]

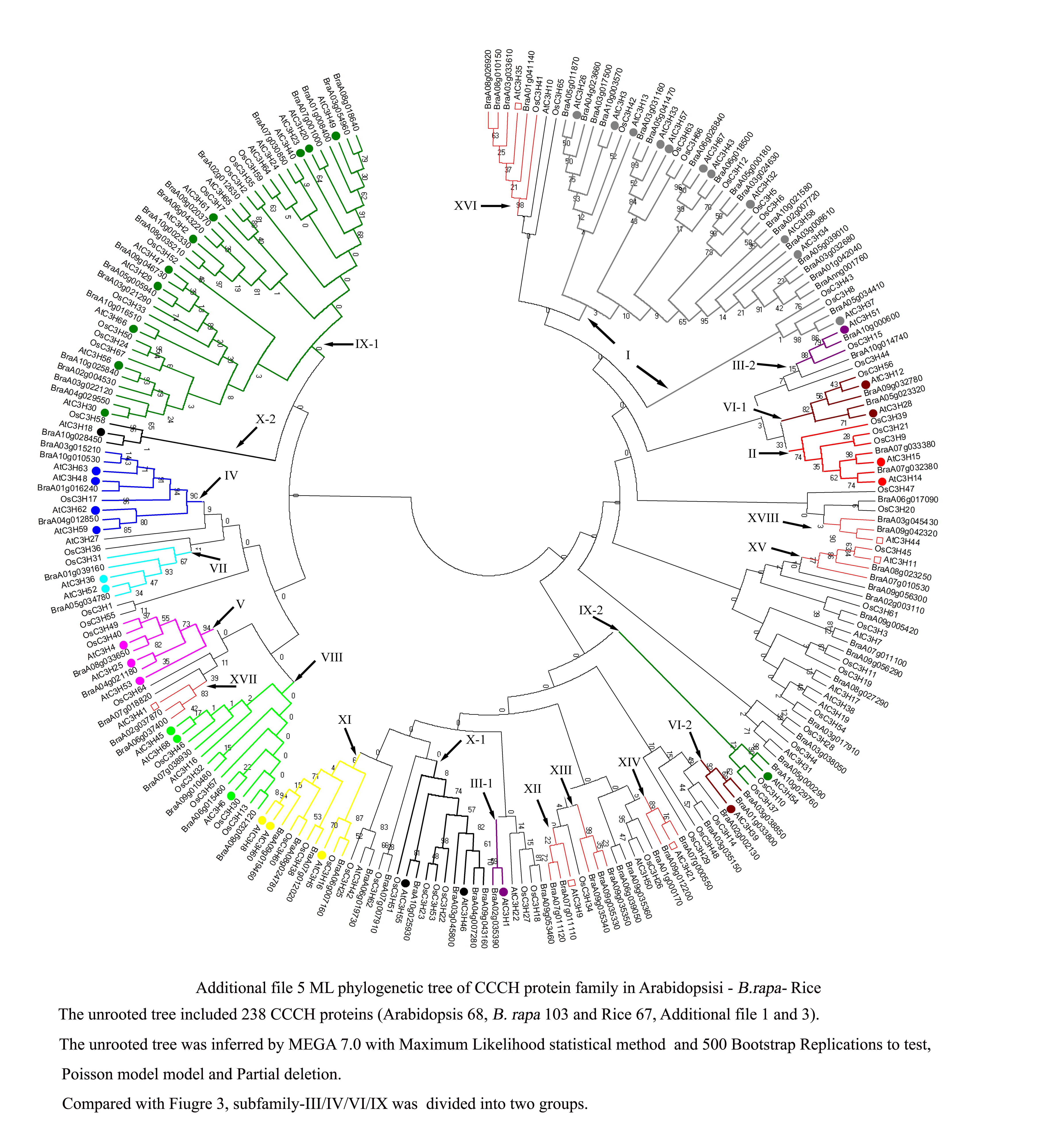

Supplement: Supplementary file 5 — ML phylogenetic tree of Arabidopsis-B. rapa-Rice. 238 CCCH proteins sequences (Arabidopsis 68, B. rapa 103, Rice, 67) were aligned using MUSCLE and the phylogenetic tree analysis was performed using MEGA 7.0. The tree was constructed with the following settings: Statistical Method as Maximum Likelihood; Include Sites as Partial deletion option for total sequence analyses; Substitution Model: Poisson model; and Bootstrap test of 500 replicates for internal branch reliability. (JPG 7157 kb) [file 12870_2018_1608_MOESM5_ESM.jpg]

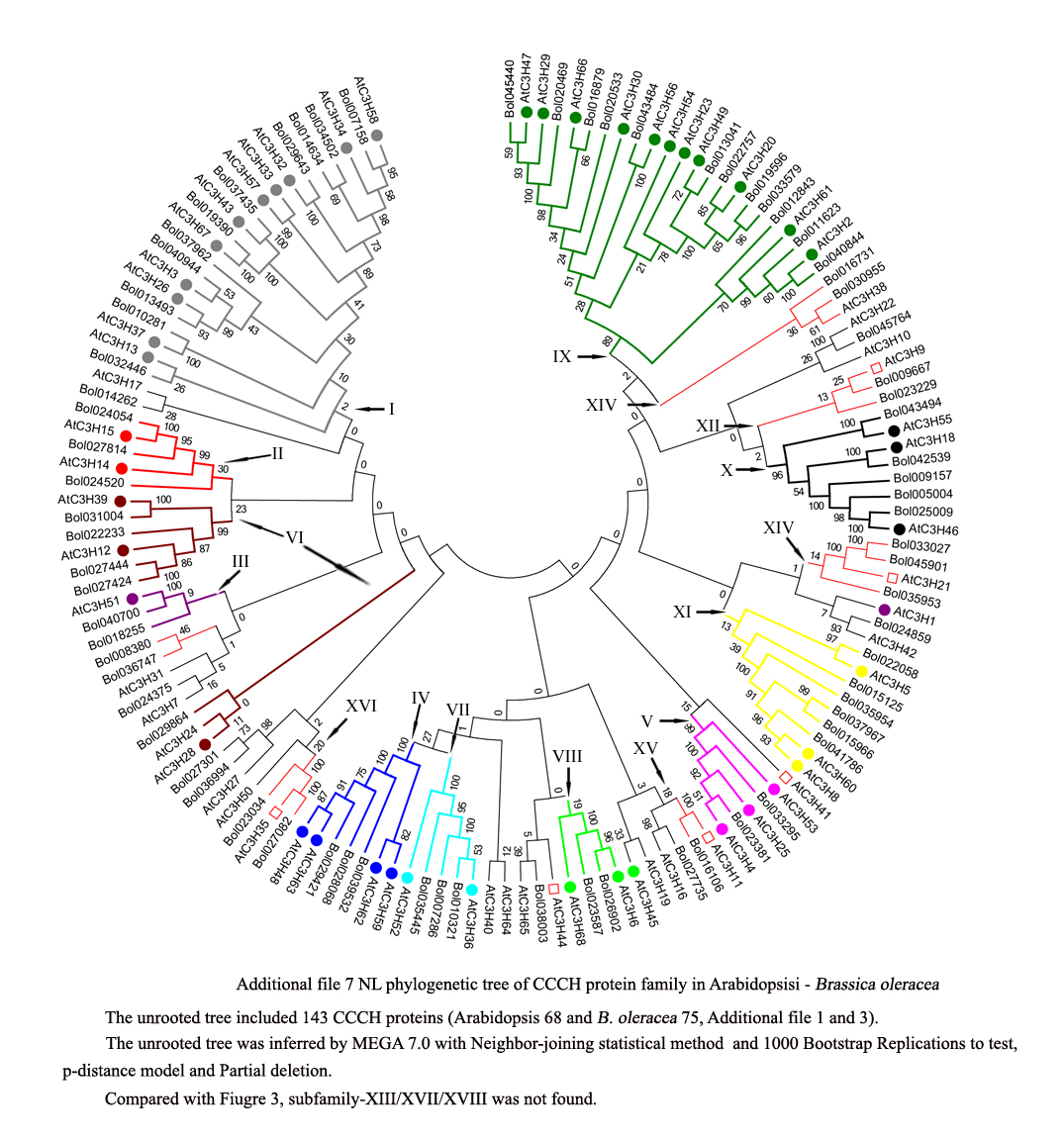

Supplement: Supplementary file 7 — NJ phylogenetic tree of Arabidopsis- B. oleracea. 143 proteins sequences (Arabidopsis 68, B. oleracea 75) were aligned using MUSCLE and the phylogenetic tree analysis was performed using MEGA 7.0. The tree was constructed with the following settings: Statistical Method as Neighbor-joining; Include Sites as Partial deletion option for total sequence analyses; Substitution Model: p-distance; and Bootstrap test of 1000 replicates for internal branch reliability. (JPG 623 kb) [file 12870_2018_1608_MOESM7_ESM.jpg]

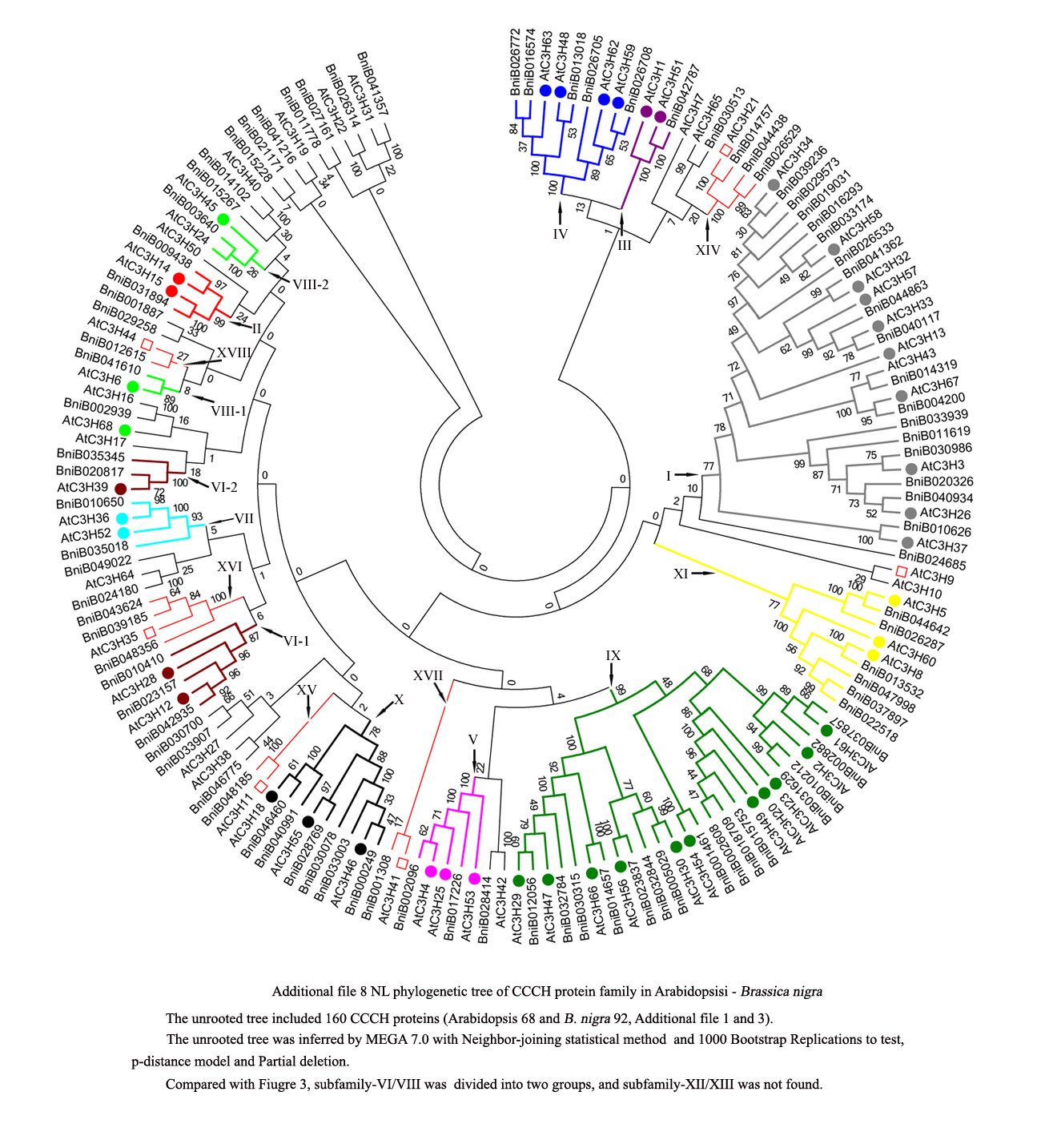

Supplement: Supplementary file 8 — NJ phylogenetic tree of Arabidopsis- B. nigra. 160 proteins sequences (Arabidopsis 68, B. nigra 92) were aligned using MUSCLE and the parameter of phylogenetic tree analysis was same to additional file 7. (JPG 831 kb) [file 12870_2018_1608_MOESM8_ESM.jpg]
